# Editorial comment: Periprocedural antiplatelet therapy during mechanical thrombectomy: navigating limited evidence

**DOI:** 10.1093/esj/aakag125

**Published:** 2026-10-19

**Authors:** Paolo Candelaresi, Antonio De Mase, Vincenzo Andreone

**Affiliations:** UOC Neurologia e Stroke Unit, AORN Cardarelli, Naples, Italy; UOC Neurologia e Stroke Unit, AORN Cardarelli, Naples, Italy; UOC Neurologia e Stroke Unit, AORN Cardarelli, Naples, Italy

**Keywords:** acute ischemic stroke, antiplatelet therapy, cangrelor, endovascular treatment, mechanical thrombectomy

The rapid evolution of endovascular stroke treatment has required clinicians to make therapeutic decisions where randomised evidence is limited or absent. Periprocedural antiplatelet therapy is a particularly challenging example. Tandem occlusions, intracranial atherosclerotic disease, rescue angioplasty and emergent stenting may require immediate platelet inhibition, yet the optimal agent, dose, combination and duration remain uncertain. In this setting, cangrelor has gained interest because of its rapid onset, potent platelet inhibition and rapid offset of action, making it attractive when prevention of acute thrombosis or reocclusion must be balanced against intracranial haemorrhage. However, increasing adoption in clinical practice should not be confused with establishment of a standard of care. Evidence supporting cangrelor in acute stroke remains predominantly observational,^1^ and pharmacological plausibility and widespread adoption do not constitute randomised evidence of superiority over alternative antiplatelet strategies.^2^

In the accompanying study, Milnerowicz and colleagues^3^ report real-world data from 644 patients receiving intraoperative cangrelor during mechanical thrombectomy across 34 French stroke centres. They compare cangrelor monotherapy with cangrelor combined with intravenous aspirin and report no statistically significant differences in functional, angiographic or safety outcomes after propensity-score overlap weighting. Importantly, the authors acknowledge that these findings cannot establish equivalence between the 2 strategies.

The study nevertheless illustrates a challenge inherent to observational comparisons of evolving therapeutic practices. The relevant clinical question is not simply whether aspirin should be added to cangrelor, but first whether cangrelor represents the optimal foundation for periprocedural platelet inhibition, in which patients, and for which endovascular indications. By design, all patients received cangrelor; the study therefore compares 2 cangrelor-based strategies rather than alternative approaches to periprocedural antiplatelet treatment.

Treatment selection is another important consideration. Aspirin administration was left to operator discretion, and patients receiving aspirin more frequently underwent adjunctive stenting and presented with tandem occlusions, dissections and large-artery atherosclerosis. Intravenous thrombolysis was also more frequent among patients receiving adjunctive aspirin. These patterns probably reflect individualised operator decisions balancing perceived thrombotic and haemorrhagic risks, a classic setting for confounding by indication. Propensity-score methods improve comparability for measured characteristics, but cannot reproduce random allocation or account for unmeasured determinants of therapeutic choice.

Clinical heterogeneity further complicates interpretation. Extracranial carotid stenting, intracranial atherosclerotic disease, refractory occlusion and rescue intracranial stenting involve different mechanisms of reocclusion and potentially different thrombotic–haemorrhagic trade-offs. An average treatment effect may conceal clinically relevant effects within particular patient groups. Rather than identifying a single optimal antiplatelet regimen for “thrombectomy patients”, future studies should determine which strategy best matches the mechanism responsible for thrombosis or reocclusion in specific endovascular scenarios.

Additional uncertainty arises from non-standardised cangrelor dosing and infusion duration and from the subsequent transition to oral antiplatelet therapy. This transition is integral to the antiplatelet strategy given cangrelor’s rapid offset and pharmacodynamic differences between oral agents.^4^ The authors acknowledge that information regarding the timing and choice of oral antiplatelet therapy was unavailable. Different transition regimens may alter platelet inhibition, potentially affecting early reocclusion, stent patency and bleeding. This is particularly relevant when day-1 arterial patency is an outcome. Early events may therefore reflect not only intraoperative cangrelor with or without aspirin but also subsequent antiplatelet management.

Perhaps most importantly, the authors appropriately emphasise that failure to detect a statistically significant benefit from adjunctive aspirin should not be interpreted as demonstrating the absence of such benefit. In an observational study not designed to assess equivalence or non-inferiority, the absence of a statistically significant difference cannot establish either. The unexpected lower rate of symptomatic intracranial haemorrhage with aspirin after excluding patients receiving prestroke antiplatelet therapy, appropriately interpreted cautiously by the authors, further illustrates rather than resolves this uncertainty.

Large multicentre registries remain essential for describing contemporary practice, identifying safety signals and generating hypotheses. Within this selected population, the study did not identify statistically significant differences in the examined outcomes between cangrelor alone and cangrelor combined with intravenous aspirin. These findings do not demonstrate that aspirin is unnecessary, that cangrelor monotherapy is equivalent to combined therapy, or that cangrelor itself should constitute the reference periprocedural antiplatelet strategy.

Future studies should move beyond comparisons of clinician-selected treatments towards prospective evaluation of standardised antiplatelet strategies, ideally through randomised allocation. Until then, this study should be viewed as an important description of what happens when cangrelor is used, rather than evidence defining when cangrelor should be used or what should accompany it. In rapidly evolving stroke intervention, clinical practice will inevitably sometimes precede randomised evidence. The challenge is to ensure that widespread practice does not itself become a substitute for evidence.

## Data Availability

Data availability is not applicable to this article as no new data were created or analysed.
